# Neurodevelopmental profile of Fetal Alcohol Spectrum Disorder: A systematic review

**DOI:** 10.1186/s40359-017-0191-2

**Published:** 2017-06-23

**Authors:** Shannon Lange, Joanne Rovet, Jürgen Rehm, Svetlana Popova

**Affiliations:** 1 Institute for Mental Health Policy Research, Centre for Addiction and Mental Health , Toronto, ON Canada; 2 Institute of Medical Science, University of Toronto, Toronto, ON Canada; 3 Neuroscience and Mental Health Program, The Hospital for Sick Children, Toronto, ON Canada; 4 Department of Pediatrics, University of Toronto, Toronto, ON Canada; 5 Dalla Lana School of Public Health, University of Toronto, Toronto, ON Canada; 6 Institute of Clinical Psychology and Psychotherapy & Center of Clinical Epidemiology and Longitudinal Studies, Technische Universität Dresden, Dresden, Germany; 7 Factor-Inwentash Faculty of Social Work, University of Toronto, Toronto, ON Canada

**Keywords:** Classification accuracy, Fetal Alcohol Spectrum Disorder, Neurodevelopmental profile, Prenatal alcohol exposure, Systematic review

## Abstract

**Background:**

In an effort to improve the screening and diagnosis of individuals with Fetal Alcohol Spectrum Disorder (FASD), research has focused on the identification of a unique neurodevelopmental profile characteristic of this population. The objective of this review was to identify any existing neurodevelopmental profiles of FASD and review their classification function in order to identify gaps and limitations of the current literature.

**Methods:**

A systematic search for studies published up to the end of December 2016 reporting an identified neurodevelopmental profile of FASD was conducted using multiple electronic bibliographic databases. The search was not limited geographically or by language of publication. Original research published in a peer-reviewed journal that involved the evaluation of the classification function of an identified neurodevelopmental profile of FASD was included.

**Results:**

Two approaches have been taken to determine the pathognomonic neurodevelopmental features of FASD, namely the utilization of i) behavioral observations/ratings by parents/caregivers and ii) subtest scores from standardized test batteries assessing a variety of neurodevelopmental domains. Both approaches show some promise, with the former approach (which is dominated by research on the Neurobehavioral Screening Tool) having good sensitivity (63% to 98%), but varying specificity (42% to 100%), and the latter approach having good specificity (72% to 96%), but varying sensitivity (60% to 88%).

**Conclusions:**

The current review revealed that research in this area remains limited and a definitive neurodevelopmental profile of FASD has not been established. However, the identification of a neurodevelopmental profile will aid in the accurate identification of individuals with FASD, by adding to the armamentarium of clinicians. The full review protocol is available in PROSPERO (http://www.crd.york.ac.uk/PROSPERO/); registration number CRD42016039326; registered 20 May 2016.

## Background

Fetal Alcohol Spectrum Disorder (FASD) is a term that encompasses a range of disorders, all of which involve prenatal alcohol exposure as the etiological cause. The effects of prenatal alcohol exposure can vary from mild to severe, and can include a broad array of cognitive, behavioral, emotional, adaptive functioning deficits, as well as congenital anomalies. FASD includes the following alcohol-related diagnoses: Fetal Alcohol Syndrome (FAS), Partial FAS (pFAS), Alcohol-Related Neurodevelopmental Disorder (ARND), and depending on the diagnostic guideline, Alcohol-Related Birth Defects (ARBD; [1, 2]). Recently, it has been proposed that FASD be used as a diagnostic term with the specification of the presence or absence of the sentinel facial features, rather than simply a non-diagnostic umbrella term [3]. This is in line with the Diagnostic and Statistical Manual of Mental Disorders, Fifth Edition (DSM-5; [4]) where Neurobehavioral Disorder Associated with Prenatal Alcohol Exposure (ND-PAE) was included as a condition that warrants further research and also as one specifier for the broader diagnostic term of Other Specified Neurodevelopmental Disorder. ND-PAE is intended to encompass the behavioral, developmental and mental health symptoms associated with prenatal alcohol exposure and is appropriate for individuals with or without physical findings [5].

With the exception of ARBD, all of the disorders within the spectrum are associated with a broad array of neurodevelopmental deficits [6–9]. Specifically, individuals with FASD exhibit relative deficits in adaptive function, attention, executive function, externalizing behaviors, motor function, social cognition, and verbal and nonverbal learning [10, 11].

Until very recently, the specific domains of function to be evaluated during the neurodevelopmental assessment have been relatively undefined and have lacked consensus [12]. The diagnostic guidelines have had a tendency to focus on the severity of the neurodevelopmental impairments rather than the specificity of the impairments. This weakness of the former diagnostic guidelines mainly impacted the diagnosis of ARND, given that diagnosis is based primarily on the neurodevelopmental impairments the child exhibits as the characteristic facial traits and growth deficits associated with FAS and pFAS are often absent with ARND. Yet, ARND is recognized to be the largest category of affected individuals, representing as many as 80–90% of FASD cases [13]. In addition to the ambiguity surrounding the diagnosis of FASD, the neurodevelopmental assessment is thought to be the lengthiest and most cumbersome component of the diagnostic evaluation [14]. Following the revised clinical guidelines of Hoyme and colleagues [2] and the proposed criteria for ND-PAE [5], three primary domains of functional impairment have been identified, namely neurocognition, self-regulation and adaptive functioning. Nevertheless, more information is needed regarding the validity of the available diagnostic approaches and the suggested cut-points.

Further, coupled with the fact that the signs of such conditions as traumatic head injury and intellectual disability where the etiological cause is not prenatal alcohol exposure are similar to FASD, the diagnostic criteria of FASD may also overlap with other neurodevelopmental disorders such as Attention Deficit Hyperactivity Disorder (ADHD), Oppositional Defiant Disorder (ODD), and Conduct Disorder (CD) [15]. As a result, individuals with FASD often receive multiple diagnoses before actually being assessed for and diagnosed with FASD [16]. It is important to note that diagnostic misclassification can have a number of untoward consequences, particularly inappropriate treatments and interventions, mismanagement of behavioral symptoms, inaccurate incidence and prevalence estimates, and reduced ability to detect a significant difference between diagnostic groups in clinical research studies [16, 17].

Therefore, in an effort to improve the screening and diagnosis of individuals with FASD, most research to date has focused on the identification of a distinct neurodevelopmental profile of FASD – defined as the outward expression (behavioral and developmental) of the central nervous system damage caused by prenatal alcohol exposure. The notion that a distinctive neurodevelopmental profile exists in individuals with FASD first emerged in the late 1990s by Stressiguth and colleagues [18]. However, identifying a neurodevelopmental profile remains to be a challenge given the wide range of deficits individuals with FASD exhibit, as well as the fact that their deficits may overlap with other neurodevelopmental disorders. Moreover, in order to determine how well a profile can accurately identify individuals with FASD, it must be tested in a diverse population and also be both sensitive and specific.^1^

In order to identify gaps and limitations of the existing literature, the current review aimed to i) identify existing neurodevelopmental profiles of FASD and ii) review the classification function (the ability of a profile to determine to which group each case most likely belongs – i.e., the sensitivity and specificity) of the respective profiles. As such, the current review is limited to those profiles for which their classification function, as a binary classification test, has been evaluated.

## Methods

### Comprehensive systematic literature search

The systematic literature search was conducted and reported according to the standards set out in Preferred Reporting Items for Systematic Reviews and Meta-Analyses [19]. A systematic literature search was performed to identify all studies that have identified a neurodevelopmental profile of FASD and were published between November 1, 1973, when FAS was first described [20], and December 30, 2016. The search was conducted in multiple electronic bibliographic databases, which included: CINAHL, Embase, ERIC, Medline, Medline in process, PsychINFO, Scopus and Web of Science (including Arts and Humanities Citation Index, Science Citation Index, and Social Sciences Citation Index). The following key words were used: 1) alcohol* embryopath*, alcohol* related* neurodevelopmental* disorder*, alcohol* related* birth defect*, arnd, arbd, fetal* alcohol* effect*, fae, fas, fasd, fetal alcohol syndrome*, fetal alcohol spectrum disorder*, foetal* alcohol* effect, foetal* alcohol syndrome*, foetal* alcohol spectrum disorder*, pfas, partial fetal alcohol syndrome, partial foetal alcohol syndrome, prenatal* alcohol expos*, OR pre-natal* alcohol expos*; AND 2) behavio*, cogniti*, development*, neurobehavio*, neurocogniti*, neurodevelopment*, neuropsycholog*, OR psycholog*; AND 3) profile*, phenotype*, OR profile analysis. The search was not limited geographically or by language of publication. Manual reviews of the content pages of the major journals in the field of neurodevelopmental disorders were conducted, as well as citations in any of the relevant articles. The full review protocol is available in PROSPERO (http://www.crd.york.ac.uk/PROSPERO/), registration number CRD42016039326.

### Inclusion and exclusion criteria

Articles were included if they were full-text articles (i.e., conference abstracts were excluded) consisting of original, quantitative research published in a peer-reviewed journal that identified a neurodevelopmental profile of FASD. Articles were excluded if they did not involve an evaluation of the classification function of the identified neurodevelopmental profile of FASD.

### Data selection and extraction

Study selection began by screening titles and abstracts for inclusion. Then, full-text articles of all studies screened as potentially relevant were considered. All data were extracted by one investigator and then independently crosschecked by a second investigator for accuracy against the original studies. All discrepancies were reconciled by team discussion.

### Uncertainty

In order to estimate the level of uncertainty surrounding the classification estimates, exact 95% confidence intervals (CI) were estimated using a binomial distribution.

## Results

**Fig. 1:**
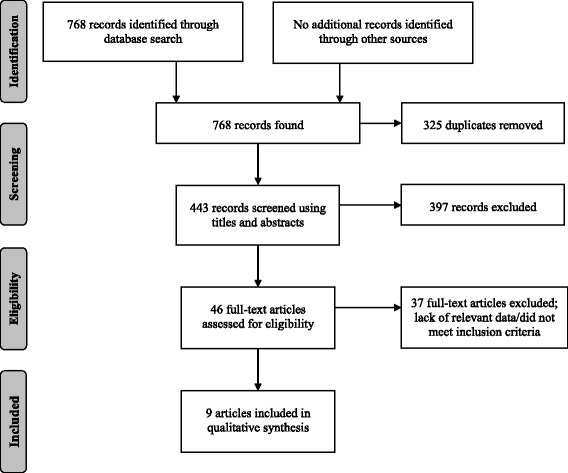
Schematic diagram depicting the search strategy employed

Initially, the search strategy yielded a total of 768 records. After removing 325 duplicates, a total of 443 records were screened using titles and abstracts. Forty-six full-text articles were retrieved for further consideration, 37 of which were subsequently excluded. This left a total of nine studies, all in English, that met the inclusion criteria and were retained for review. A schematic diagram of the search strategy is depicted in Fig. 1.

Based on the identified studies, two general approaches were observed for determining the pathognomonic neurodevelopmental features of FASD, namely: i) behavioral observations/ratings by parents/caregivers (six studies), and ii) subtest scores from standardized test batteries assessing a variety of neurodevelopmental domains (three studies).

### Neurodevelopmental profiles of FASD based on behavioral observations/ratings by parents/caregivers

The Child Behavior Checklist (CBCL; five studies) and the Behavior Rating Inventory of Executive Function (BRIEF; one study) have been used to identify a neurodevelopmental profile characteristic of FASD.

### Child Behavioral Checklist (CBCL)

Nash and colleagues [21] sought to determine if a behavioral profile distinguishes children with FASD (diagnosed according to the 2005 Canadian diagnostic guidelines; [1]) from typically developing children and children with ADHD. The CBCL is a well-established standardized parent/caregiver questionnaire utilized for evaluating social competencies and behavioral problems in children 6 to 18 years of age, and is comprised of a series of open ended questions and a rating scale of 113 behavioral descriptors. The authors utilized discriminant function analysis and Receiver Operating Characteristics curve analyses to determine sensitivity and specificity of different item combinations. Findings revealed ten specific behavioral characteristics captured by the CBCL (Table 1) had the potential to differentiate between children with FASD from children with ADHD and typically developing control children, all 6 to 16 years of age. Specific item combinations (Table 2) resulted in 86% (95% CI: 77%–95%) sensitivity and 82% (95% CI: 72%–92%) specificity when children with FAS where compared to typically developing control children, and 70% (95% CI: 58%–82%) to 81% (95% CI: 71%–91%) sensitivity and 72% (95% CI: 61%–83%) to 80% (95% CI: 70%–90%) specificity when children with FAS where compared to children with ADHD.

**Table 1:** Neurobehavioral Screening Tool (NST)

Items
1. Has your child been seen or accused of or thought to have acted too young for his or her age?
2. Has your child been seen or accused of or is thought to be disobedient at home?
3. Has your child been seen or accused of or is thought to lie or cheat?
4. Has your child been seen or accused of or is thought to lack guilt after misbehaving?
5. Has your child been seen or accused of or is thought to have difficulty concentrating, and can’t pay attention for long?
6. Has your child been seen or accused of or is thought to act impulsively and without thinking?
7. Has your child been seen or accused of or is thought to have difficulty sitting still, is restless or hyperactive?
8. Has your child been seen or accused of or is thought to display acts of cruelty, bullying or meanness to others?
9. Has your child been seen or accused of or is thought to steal items from home?
10. Has your child been seen or accused of or is thought to steal items from outside of the home?

Source: Nash and colleagues [21, 22]

*Note.* Each item has a response option of ‘Yes’ or ‘No’

**Table 2:** Classification accuracy of the Neurobehavioral Screening Tool reported in the individual studies

Reference	Age range (years)	Comparison	Items endorsed	Sensitivity	95% CI^a^		Specificity	95% CI^a^	
					Lower	Upper		Lower	Upper
Breiner et al. [23]	4 to 6	FASD (*n* = 17) vs. Deferred/Controls (*n* = 43)^b^	≥5 items (items 1, 2, 4–8; acts young, disobedient, lacks guilt, difficulty concentrating, impulsivity, hyperactive, cruelty)^c^	94%	88%	100%	96%	91%	100%
Haynes et al. [26]	6 to 12	Children born to and reared by mothers with depression (*n* = 49) vs. Controls (*n* = 22)	≥6 items (out of items 1–7; acts young, disobedient, lie/cheat, lacks guilt, difficulty concentrating, impulsivity, hyperactive) OR ≥3 items (out of items 1, 2, 3, and 4; acts young, disobedient, lie/cheat, lacks guilt)	-	-	-	100%	-	-
LaFrance et al. [24]	6 to 17	FASD (*n* = 48) vs. Controls (*n* = 32)	≥6 items (out of items 1–7; acts young, disobedient, lie/cheat, lacks guilt, difficulty concentrating, impulsivity, hyperactive) OR ≥3 items (out of items 1, 2, 3, and 4; acts young, disobedient, lie/cheat, lacks guilt)	63%	53%	74%	100%	-	-
		Children prenatally exposed to alcohol who did not meet the criteria for an FASD diagnosis (*n* = 22) vs. Controls (*n* = 32)	≥6 items (out of items 1–7; acts young, disobedient, lie/cheat, lacks guilt, difficulty concentrating, impulsivity, hyperactive) OR ≥3 items (out of items 1, 2, 3, and 4; acts young, disobedient, lie/cheat, lacks guilt)	50%	37%	63%	100%	-	-
Nash et al. [21]	6 to 18	FASD (*n* = 30) vs. Controls (*n* = 30)	≥6 items (out of items 1–7; acts young, disobedient, lie/cheat, lacks guilt, difficulty concentrating, impulsivity, hyperactive)	86%	77%	95%	82%	72%	92%
		FASD (*n* = 30) vs. ADHD (*n* = 30)	≥2 items (out of items 1, 4, and 8; acts young, lacks guilt, cruelty)	70%	58%	82%	80%	70%	90%
			≥3 items (out of items 1, 4, 8, 9, and 10; acts young, lacks guilt, cruelty, steals from home, steals from outside home)	81%	71%	91%	72%	61%	83%
Nash et al. [22]	6 to 18	FASD (*n* = 56) vs. Controls (*n* = 53)	≥3 items (out of all 10 items; acts young, disobedient, lie/cheat, lacks guilt, difficulty concentrating, impulsivity, hyperactive, cruelty, steals from home, steals from outside home)	98%	95%	100%	42%	33%	51%
		FASD (*n* = 56) vs. ADHD (*n* = 50)	≥2 items (out of items 1, 4, 8, 9, and 10; acts young, lacks guilt, cruelty, steals from home, steals from outside home)	89%	83%	95%	42%	33%	51%
		FASD (*n* = 56) vs. ODD/CD (*n* = 61)	1 item (item 1; acts young)	-	-	-	-	-	-

*ADHD* Attention Deficit Hyperactivity Disorder, *CD* Conduct Disorder, *CI* Confidence Interval, *FASD* Fetal Alcohol Spectrum Disorder, *ODD* Oppositional Defiant Disorder

^a^Estimated by the current author, using a binomial distribution

^b^It is assumed that children with FASD were compared to children for whom a diagnosis could not be confirmed or was deferred in combination with control children (methods and results sections were inadequate to determine if this assumption is correct)

^c^Items 3, 9, and 10 (lie/cheat, steal at home, and steal outside the home) were excluded from the analysis due to the inability to verify these items in most young children

Nash, Koren, and Rovet [22] replicated their earlier study [21] using a larger sample and comparing children with FASD (diagnosed according to the 2005 Canadian Guidelines; [1]) to children with ODD/CD, as well as children with ADHD and typically developing control children in order to establish the specificity of the 10-item screening tool. All children ranged in age from 6 to 18 years of age. Findings revealed the tool differentiated children with FASD from control children with 98% (95% CI: 95%–100%) sensitivity and 42% (95% CI: 33%–51%) specificity, and from children with ADHD with 89% (95% CI: 83%–95%) sensitivity and 42% (95% CI: 33%–51%) specificity. However, sensitivity and specificity could not be determined for discriminating children with FASD from children with ODD/CD since only one item significantly differentiated these groups, namely “acts young”.

From their preliminary investigations showing that certain behaviors had the potential to identify children with a high likelihood of having FASD, Nash and colleagues [21, 22] proposed using this 10-item questionnaire as a screening tool and coined it the “Neurobehavioral Screening Tool (NST)”. Based on the two studies discussed above [21, 22], it was discerned that the NST has the potential to delineate children with FASD from children with ADHD and normally developing children. However, these two studies were limited in that they retrospectively extracted items from the fully administered CBCL, and their samples consisted of children aged 6 to 18 only. The former limitation is noteworthy given that the CBCL is scored on a three-point scale (i.e., “not true”, “somewhat or sometimes true”, and “very true or often true”); the authors of the NST collapsed the responses “somewhat or sometimes true” and “very true or often true” and this can affect the classification accuracy. The latter limitation means that the behaviors noted in the NST cannot be assumed to be reflective of children with FASD outside this age range (i.e., less than 6 and over 18 years of age).

Accordingly, Breiner, Nulman, and Koren [23] conducted a study in order to determine if the NST could be validated among a sample of children diagnosed with FASD (according to the 2005 Canadian Guidelines; [1]), children with either a deferred diagnosis or for whom a diagnosis could not be confirmed, and normally developing control children, all 4 to 6 years of age. Three items (lie/cheat, steal at home, and steal outside the home) were excluded from the analysis due to the inability to verify these items in most young children. Using the seven remaining items, the authors found that the NST had 94% (95% CI: 88%–100%) sensitivity and 96% (95% CI: 91%-100%) specificity in identifying children with FASD (Table 2). However, it is unclear from which group children with FASD were discriminated (i.e., if the non-diagnosed group was combined with the control children), as the methods and results sections describing it are inadequate. Further, this study retrospectively extracted items from the CBCL in its entirety.

More recently, LaFrance et al. [24] administered the NST as a stand-alone instrument to parents/caregivers of children 6 to 17 years of age and thus, addressed the limitation of collapsing items originally scored on a three-point scale [21–23]. Using the scoring approach published by Nash and associates [21], compared with normally developing control children, the NST yielded 63% (95% CI: 52%–74%) sensitivity and 100% (not possible to estimate 95% CI) specificity for children with FASD (diagnosed according to the 4-Digit Diagnostic Code; [25]) and 50% (95% CI: 37%–63%) sensitivity and 100% (not possible to estimate 95% CI) specificity for children prenatally exposed to alcohol who did not meet the diagnostic threshold when assessed (Table 2). This study also assessed possible age- and sex-related differences on the NST, by comparing 6–to 11-year old children with 12–to 17-year old adolescents, and boys versus girls. For both the FASD group and the group of children prenatally exposed to alcohol who did not meet the diagnostic threshold, the NST showed higher sensitivity among adolescents (71% [95% CI: 61%–81%] and 71% [95% CI: 59%–83%], respectively) when compared with children (54% [95% CI: 43%–65%] and 40% [95% CI: 27%–53%], respectively). For the FASD group only, the NST also had higher sensitivity among boys when compared with girls (71% [95% CI: 61%–81%] and 56% [95% CI: 45%–67%], respectively). Specificity was found not to differ with respect to age and sex, as it was 100% (not possible to estimate 95% CI) in all of the comparisons. Lastly, the authors explored an alternative cumulative scoring option, with the endorsement of at least four items resulting in 90% (95% CI: 83%–97%) sensitivity and 91% (95% CI: 85%–97%) specificity. This study is not only the first to administer the NST as a stand-alone instrument, but is also the first to differentiate children prenatally exposed to alcohol who do not meet the criteria for an FASD diagnosis from typically developing control children. The discrimination of children prenatally exposed to alcohol who did not meet the criteria for an FASD diagnosis helps to further establish the specificity and discriminate validity of the NST. Nonetheless, it must be noted that this study involved the retrospective administration of the NST in a sample of children that had had already undergone a full diagnostic evaluation, thereby limiting the degree to which the results can be said to establish the validity of the NST as a “screening” tool per se.

In order to further establish the specificity of the NST, Haynes, Nulman, and Koren [26] recently evaluated the influence of maternal depression – the most prevalent psychiatric morbidity among women with difficulties inhibiting their consumption of alcohol during pregnancy [27] – on the previously identified behavioral presentation of children with FASD [21, 22, 24] (diagnosed according to either the 2005 Canadian diagnostic guidelines [1] or the 4-Digit Diagnostic Code [25]). Specifically, the investigators sought to determine if the NST resulted in any false positives among a sample of children born to and reared by mothers with clinical depression and typically developing control children. None of the children with mothers suffering from depression scored positive on the NST (100% specificity, not possible to estimate 95% CI; Table 2). In fact, only one item (hyperactive) was found to be significantly higher in the group of children with mothers suffering from depression, compared with the control children.

In summary, the NST has demonstrated good sensitivity (63% to 98%), but varying specificity (42% to 100%, with some estimates being unfavorably low), and thus should still be considered in the validation stage. It is important to note that the NST is intended for screening purposes only [21, 22], and given it is limited to overt behaviors only, its ability as a diagnostic tool is questionable since it does not fully capture all neurodevelopmental impairments seen among individuals with FASD. However, there are few limitations of the available studies on the NST that should be noted. First, all of the studies evaluating the psychometric utility of the NST are plagued by small or modest at best, clinically-referred Canadian samples, thus limiting generalizability of the above findings. Second, the NST has the inherent problem of providing the behavioral observations of parent or parent substitutes, who by definition are not masked to the child’s history and thus may convey observations distorted by positive intent. Third, although a few of the studies investigating the NST specified whether the participants that made up the comparison groups were screened for prenatal alcohol exposure, and subsequently excluded [21, 22], others did not [23, 24, 26].

### Behavior Rating Inventory of Executive Function (BRIEF)

Recently, Nguyen and colleagues [28] sought to determine whether the BRIEF clinical scales, a parent/caregiver questionnaire that consists of 86-items and eight empirically derived clinical scales assessing executive function and self-regulation in children 5 to 18 years of age, can distinguish among the following four groups of children: 79 children prenatally alcohol-exposed with ADHD; 36 children prenatally alcohol-exposed without ADHD; 90 children with idiopathic ADHD (without prenatal alcohol exposure); and 168 typically developing control children. Prenatal alcohol exposure was defined as at least four drinks per occasion at least once per week or at least 14 drinks per week during pregnancy. A discriminant function analysis revealed that the following four clinical scales best distinguished the groups: i) Inhibit, which describes a child’s ability to tune out irrelevant stimuli; ii) Emotional Control, which describes a child’s ability to modulate emotional responses; iii) Working Memory, which describes a child’s ability to hold information in mind for the purpose of completing a task; and iv) Organization of Materials, which describes a child’s orderliness of work, play, and storage spaces. Classification accuracy was 71% (95% CI: 66%–76%) overall, with 67% (95% CI: 62%–72%) of children prenatally alcohol-exposed with ADHD, 43% (95% CI: 38%–48%) children prenatally alcohol-exposed without ADHD, 51% (95% CI: 46%–56%) of children with idiopathic ADHD, and 92% (95% CI: 89%–95%) of typically developing control children classified correctly.

Although its use as tool to discriminate individuals with FASD from other clinical populations is still in the exploratory stages, the BRIEF appears to distinguish alcohol-exposed children with ADHD from those with idiopathic ADHD, and thus may be useful as a screening tool. However, based on the results presented above, the ability of the BRIEF to identify children prenatally alcohol-exposed without ADHD is limited.

### Neurodevelopmental profiles of FASD based on subtest scores from a battery of standardized tests

Mattson and colleagues [29] sought to identify a neurodevelopmental profile of FASD using subtest scores from a battery of neurodevelopmental tests administered to individuals heavily exposed to alcohol prenatally, defined as four or more drinks per occasion at least once per week or 13 or more drinks per week, and individuals with no prenatal alcohol exposure or minimal exposure, defined as no more than one drink per week on average and a maximum of two drinks per occasion. All participants were between 7 and 21 years of age and subsequently categorized based only on physical features, regardless of their exposure status. Classifications included “FAS”, defined as the presence of at least two of the three key facial features (short palpebral fissures, smooth philtrum, and thin vermillion boarder) and either microcephaly (head circumference ≤10^th^ percentile) or growth deficiency (weight and/or height ≤10^th^ percentile) or both; “Not FAS”; or “Deferred”, defined as the presence of at least one key facial feature, or microcephaly and growth deficiency, or microcephaly or growth deficiency and at least one additional specified feature documented to be prevalent among those with FASD such as ptosis, and camptodactyly. Twenty-two variables, derived from the subtests of a battery of standardized tests, were selected based on their effect size in detecting the difference between exposed and unexposed individuals.

Two latent profile analyses were performed in order to derive a discriminative profile. In both analyses, a two-class solution fit better than a one-class solution – meaning that, based on the response means, it was more likely that there were two unobserved groups in the sample used in each analysis. In the first analysis, exposed individuals who met the study criteria for FAS (*n* = 41) were compared with unexposed individuals categorized as Not FAS (*n* = 46); the resulting profile had an overall classification accuracy of 92% (95% CI: 86%–98%), with 88% (95% CI: 81%–95%) sensitivity and 96% (95% CI: 92%–100%) specificity. In the second analysis, exposed individuals categorized as Not FAS or Deferred (*n* = 38) were compared with unexposed individuals categorized as Not FAS or Deferred (*n* = 60); the resulting profile had an overall classification accuracy of 85% (95% CI:78%–92%), with 68% (95% CI: 59%–77%) sensitivity and 95% (95% CI: 91%–99%) specificity. The discriminative profile consisted of deficits in executive function, attention, spatial reasoning and memory, fine motor speed, and visual motor integration (Table 3). In both analyses, individuals categorized as belonging to “Group 1” performed more poorly than those belonging to “Group 2”, with significantly more alcohol-exposed individuals in “Group 1” and significantly more unexposed individuals in “Group 2”. See Table 3 for the measures included in the profile and neurodevelopmental domains assessed.

**Table 3:** Measures included in the profile and neurodevelopmental domains assessed by Mattson and colleagues [29]

Observed variable/measure	Neurodevelopmental domain(s) measured
CANTAB Spatial Recognition Memory Percent Correct (z-score)	Visual memory, spatial reasoning
CANTAB Spatial Span Length (z-score)	Executive function, spatial reasoning, visual memory
CANTAB Spatial Working Memory Strategy (z-score)	Executive function, spatial working memory
CANTAB Spatial Working Memory Total Errors (z-score)	Executive function, spatial working memory
D-KEFS Trail Making Combined Number/Letter (scaled score)	Executive function, sequencing
D-KEFS Trail Making–Switch versus Number (scaled score)	Executive function, cognitive flexibility
D-KEFS Trail Making–Switch versus Visual (scaled score)	Executive function
D-KEFS Trail Making–Switch Errors (scaled score)	Executive function, cognitive flexibility
D-KEFS Verbal Fluency Total Correct Letter (scaled score)	Executive function, fluency
D-KEFS Verbal Fluency Total Correct Category (scaled score)	Executive function, fluency
D-KEFS Verbal Fluency Total Correct Switch (scaled score)	Executive function, cognitive flexibility
D-KEFS Verbal Fluency Second Interval Correct (scaled score)	Executive function, fluency
D-KEFS Verbal Fluency Set Loss Errors (scaled score)	Executive function, set maintenance
MVWM Time in Target Quadrant on Probe Trail (raw score)	Spatial learning
NES3 Animals Following subtest, Number Correct (raw score)	Sustained attention
NES3 Animals Repeating subtest, Number Correct (raw score)	Sustained attention
NES3 Animals Single subtest, Number Correct (raw score)	Sustained attention
Grooved Pegboard Test Dominant Hand Completion Time (z-score)	Fine motor
Grooved Pegboard Test Non-Dominant Hand Completion Time (z-score)	Fine motor
Progressive Planning Test Maximally Constrained Total Score (raw score)	Executive function, planning
Visual Discrimination Reversal Learning Test Number of Reversals (raw score)	Executive function, cognitive flexibility
Visual Motor Integration Test Total (standard score)	Visual-motor

*CANTAB* Cambridge Neuropsychological Test Automated Battery, *D-KEFS* Delis-Kaplan Executive Function System, *MVWM* Morris Virtual Water Maze, *NES3* Neurobehavioral Evaluation System 3

In a subsequent study, Mattson and colleagues [30] attempted to further refine their initial neurodevelopmental profile [29] by i) reducing the number of variables included, ii) using a larger sample between 8 and 17 years of age, and iii) including a clinical contrast group. The same definitions of “heavily exposed to alcohol prenatally” and “no prenatal alcohol exposure or minimal exposure” were used as before [29]. Based on clinical judgment and expertise, researchers selected 11 variables from the large test battery, four of which overlapped with those selected in the previous study [29] (Note: overlapping measures are indicated with an asterisk in Table 4).

**Table 4:** Measures included in the profile and neurodevelopmental domains assessed by Mattson and colleagues [30]

Observed variable/measure	Neurodevelopmental domain(s) measured
CANTAB Delayed Matching to Sample Percent Correct (z-score)	Short-term and long-term visual and spatial memory
CANTAB Intra-Extra Dimensional Shift Stages Completed (z-score)	Executive function, cognitive flexibility
CANTAB Intra-Extra Dimensional Shift Total Errors (z-score)	Executive function, cognitive flexibility
CANTAB Simple Reaction Time Percent Correct Trials (raw score)	Attention, reaction time
CANTAB Spatial Working Memory Total Errors (z score)*	Executive function, spatial working memory
D-KEFS Color-Word Interference Inhibition/Switching (scaled score)	Executive function, inhibitory control, cognitive flexibility
D-KEFS Trail Making–Switch versus Number (scaled score)*	Executive function, cognitive flexibility
D-KEFS 20 Questions Total Initial Abstraction (scaled score)	Executive function, planning, deduction
D-KEFS Tower Test Rule Violations Per Item Ratio (scaled score)	Executive function, planning
D-KEFS Verbal Fluency Total Correct Letter (scaled score)*	Executive function, fluency
D-KEFS Verbal Fluency Total Correct Switch (scaled score)*	Executive function, cognitive flexibility

*Indicates the measures that overlap with those selected in Mattson et al. [29]

*CANTAB* Cambridge Neuropsychological Test Automated Battery, *D-KEFS* Delis-Kaplan Executive Function System

Three latent profile analyses were conducted. In all three analyses, a two-class solution fit better than a one-class solution. In the first analysis, exposed individuals who met the study criteria for FAS (same criteria as the authors previous study [29]; *n* = 79) were compared with unexposed individuals (*n* = 185) and the resulting profile yielded an overall classification accuracy of 76% (95% CI: 71%–81%), with 77% (95% CI: 72%–82%) sensitivity and 76% (95% CI: 71%–81%) specificity. In the second analysis, exposed individuals who did not meet the criteria for FAS (*n* = 117) were compared with unexposed individuals (*n* = 185); the resulting profile had an overall classification accuracy of 72% (95% CI:67%–77%), with 70% (95% CI: 65%–75%) sensitivity and 72% (95% CI: 67%–77%) specificity. The third analysis comparing exposed individuals with and without FAS (*n* = 209) and individuals with ADHD who were not exposed to alcohol prenatally (as per the definition of prenatal alcohol exposure used by the authors; *n* = 74) led to a profile with an overall classification accuracy of 74% (95% CI: 69%–79%), with 60% (95% CI: 54%–66%) sensitivity and 76% (95% CI: 71%–81%) specificity. The discriminative profile consisted of deficits in executive function, attention, and visual and spatial memory, with measures of executive function most effectively distinguishing individuals prenatally alcohol-exposed from those not exposed (Table 4). In all three analyses, significantly more alcohol-exposed individuals belonged to “Group 1” and significantly more unexposed individuals to “Group 2” (see Table 4 for the measures included in the profile and neurodevelopmental domains assessed).

From a clinical perspective, the psychometric utility of the profile of Mattson and colleagues [30] was not optimal in discriminating those with FASD from those with ADHD – it was more accurate at identifying individuals with ADHD than individuals with FASD. Further, it appears that a more limited test battery is not equally as useful at distinguishing between individuals with FASD and unexposed individuals as a larger test battery, as the sensitivity was reduced from 88% in the first study [29] to 77% in the second study [30]. Lastly, although the classification rates were significant, a number of subjects were misclassified. Further, the two studies by Mattson et al. [29, 30] have a few limitations to note. First, coupled with the fact that the authors utilized test batteries that accommodated the large age range and language variations of their samples, the batteries used do not constitute a full clinical assessment battery typically used in an FASD diagnostic clinics. As such, the test batteries lacked clinical sensitivity and likely excluded other measures that may have been useful in distinguishing individuals with FASD from unexposed controls and other clinical populations. Second, the samples were made up of participants clinically referred for suspected problems or exposures and thus, prone to sampling bias, undermining the external validity of the findings. Third, the investigators only included weaknesses in their neurodevelopmental profile and did not include relative strengths. Fourth, the classification of individuals as having FAS was based on physical traits only, and is not reflective of how FAS is classified elsewhere (see for example, the Canadian guidelines for diagnosis; [1]).

Recently, Enns and Taylor [31] used logistic regression to determine which neurodevelopmental variables are most predictive of an FASD diagnosis. Studied were 180 children and adolescents (5 to 17 years of age) prenatally exposed to alcohol, 107 of whom received a diagnosis of FASD according to the 2005 Canadian diagnostic guidelines [1] and 73 who did not. The authors identified a model that incorporated specific intelligence indices (verbal intelligence and working memory), academic achievements (spelling and math calculations), auditory working memory, and spatial planning correctly classified 75% (95% CI: 70%–80%) of cases (sensitivity and specificity were not reported). However, it was not clear if scaled scores were used in the model, and the most obvious limitation of the study is that data was retrospectively collected via a chart review of a clinically referred sample. Further, given the retrospective nature of the study, the number of children and adolescents assessed using each measure varied – however, the sample size was not specified for the final profile. Although the identified profile was able to differentiate individuals diagnosed with FASD from those who were prenatally exposed to alcohol but whom did not receive a diagnosis of FASD, the ability to differentiate individuals with FASD from unexposed individuals and individuals with other clinical populations remains unclear. See Table 5 for the measures included in the profile and neurodevelopmental domains assessed by Enns and Taylor [31].

**Table 5:** Measures included in the profile and neurodevelopmental domains assessed by Enns and Taylor [31]

Observed variable/measure	Neurodevelopmental domain(s) measured
CMS Stories: Delayed/WMS-IV Logical Memory II	Auditory working memory
D-KEFS Tower: Total Achievement	Executive function, spatial planning
WISC-IV Working Memory Index	Working memory
WISC-IV Verbal Comprehension Index	Verbal intelligence
WRAT4 Math Calculations	Academic achievement, mathematical ability
WRAT4 Spelling	Academic achievement, basic reading and spelling ability

*CMS* Children’s Memory Scale, *D-KEFS* Delis-Kaplan Executive Function System, *WISC-IV* Wechsler Intelligence Scale for Children, Fourth Edition, *WMS-IV* Wechsler Memory Scale, Fourth Edition, *WRAT4* Wide Range Achievement Test, Fourth Edition

## Discussion

Based on the studies reviewed above, it is clear that a definitive neurodevelopmental profile of FASD has yet to be identified. However, the current literature has notable clinical implications. First, behavioral ratings by primary caregivers have the potential to be used in the development of a screening tool, which can be used to identify those children most in need of a full multi-disciplinary diagnostic assessment. Second, a battery of neurodevelopmental tests can be used to distinguish between children with FASD and typically developing children, children prenatally exposed to alcohol but who do not meet the criteria for a diagnosis of FASD, as well as children with ADHD. Overall, the results of the current review support a stepwise approach the diagnosis of FASD. A diagnosis of FASD has a number of important benefits namely, participation in developmental interventions, improved quality of life and a more prosperous developmental trajectory in terms of social functioning.

Although a biomarker would be the most ideal method for diagnosing cases of FASD, at this time observational data and neurodevelopmental testing are the most appropriate tools. Thus, the identification of a distinct neurodevelopmental profile that is pathognomonic of FASD will assist in the: i) accurate identification of individuals with FASD, by adding to the resources available to clinicians; ii) discrimination of FASD from other clinical populations (i.e., differential diagnosis); iii) ascertainment of accurate prevalence estimates; iv) planning/development of appropriate targeted interventions for individuals with FASD; and v) enhancement of clinical services to this population. Coupled with the fact that the neurodevelopmental assessment is both time consuming and costly [14], the current capacity of diagnostic services is also limited [32]. Thus, delineating the specific neurodevelopmental profile of FASD will not only reduce the time it takes to fully assess an individual, but it will also assist in triaging children most in need of a full clinical assessment [21, 22].

Nevertheless, studies utilizing observational and/or neurodevelopmental data to identify the presence of a unique neurodevelopmental profile of FASD are not without their limitations (e.g., confounding, and a lack of normative data with respect to FASD and mixed racial groups). In addition to the inherent data limitations, the two approaches currently used in determining the neurodevelopmental profile of FASD are both limited in scope. For instance, the approach involving observations/ratings of parents/caregivers (i.e., the NST) is solely based on problem behaviors. However, individuals with FASD have a number of other developmental impairments and behavioral manifestations that could be useful when delineating FASD from other clinical populations. Further, the neurodevelopmental profiles based on the subtest scores from a battery of standardized tests do not consider the relative strengths of individuals with FASD [11, 33].

It should also be recognized that the studies reviewed used different diagnostic guidelines for ascertaining cases of FASD. Given that it was recently reported that existing FASD diagnostic guidelines lack convergent validity and are limited in their concordance with respect to the specific diagnostic entities [34], the consequence of this variation is that the profiles are essentially classifying different groups of affected individuals. Thus, the only conceivable way to resolve this issue is for a standardized common diagnostic approach to be developed and widely accepted. Only then will we be able to identify whether a neurodevelopmental profile of FASD exists, and truly assess its classification function.

Further, given the stigmatization associated with alcohol use during pregnancy and the increased likelihood of underreporting [35], it is possible that the comparison groups of typically developing control children used in the studies reviewed may contain some children prenatally exposed to alcohol, which is possible for example in studies of Mattson and colleagues [29, 30] given their definition of prenatal alcohol exposure. Consequently, the classification function of a particular profile could in fact be more robust than observed.

Although it is clear that the identification of a neurodevelopmental profile of FASD has a number of notable benefits, at least eight areas of future research need to be addressed before a neurodevelopmental profile is defined and put into practice. The first concerns testing the profile on larger, more diverse samples, as well as in general population screening settings (i.e., among population-based samples). Second, the profile’s ability to differentiate children with FASD from other clinical populations (e.g., other than idiopathic ADHD, without prenatal alcohol exposure) needs to be determined. Third, potential gender and age differences need to be explored, and the cross-cultural utility of the profile needs to be established. Fourth, a broader, more comprehensive array of neurodevelopmental domains needs to be evaluated. Fifth is the possibility that individuals with FASD exhibit more than one neurodevelopmental profile should be explored. For example, a distinct profile could exist for each diagnostic category. Sixth, future studies need to control for adverse prenatal exposures such as maternal smoking and drug use during pregnancy, maternal and paternal psychopathologies, and postnatal experiences including abuse and neglect. Seventh is the possibility that some of the associated neurodevelopmental symptoms were inherited from parents (e.g., a math disability) and not strictly attributable to the prenatal alcohol exposure. Eighth, it is possible that individual differences in factors that influence the consequences of prenatal alcohol exposure may interfere with the identification a unique neurodevelopmental profile of FASD given that susceptibility to prenatal alcohol exposure depends on the genotype of the fetus [36] and the developmental stage at the time of exposure, and that the manifestations of abnormal development increase in frequency and degree as dosage increases (as per the principles of teratogenesis; [37, 38]). Accordingly, genetic factors/differences in fetal susceptibility to alcohol and information on dosage and timing of exposure should also be taken into consideration when identifying and validating a neurodevelopmental profile of FASD. It is likely that many of these areas of future research will only be achievable if and when large detailed datasets are developed containing data on individuals with FASD diagnosed using a common diagnostic guideline, which will allow for certain variables (e.g., experience of postnatal adversities) to be controlled for.

However, given that the outcomes of prenatal alcohol exposure depend on a number of factors (e.g., genetics, health, alcohol metabolism, polysubstance exposure, timing of exposure [39–41]), as well as the fact that FASD is associated with multiple comorbid mental disorders [42–44], it should be acknowledged that FASD may in fact have a complex phenotype and a pathognomonic neurodevelopmental profile of FASD may not exist. It is possible that FASD has a pleiotropic phenotype (i.e., one cause (prenatal alcohol exposure) results in many outcomes); if this is the case it will negate the existence of a neurodevelopmental profile unique to those with FASD.

### Strengths and limitations

The current literature review has a number of notable strengths, namely the comprehensive search strategies, strict inclusion and exclusion criteria, and the critical approach to presenting the existing neurodevelopmental profiles of FASD. However, it is important to acknowledge that this review is limited to those profiles that were accompanied by an evaluation of their classification function. Nevertheless, there are profiles that show some promise that were not eligible for inclusion in the current review (e.g., Nash et al. [8] and Stevens et al. [45]).

## Conclusions

This systematic review elucidates the need for additional well-conducted research investigating the existence of a neurodevelopmental profile of FASD. Although research in this area is limited and a definitive neurodevelopmental profile of FASD remains to be established, the benefits of identifying a pathognomonic neurodevelopmental profile are noteworthy. It is likely that a neurodevelopmental profile of FASD that includes both behavioral observations/ratings and performance-based measures of neurodevelopment will be the most comprehensive and as such, future studies should include measures covering a broad array of neurodevelopmental and behavioral domains.

## Abbreviations

ADHD: Attention Deficit Hyperactivity Disorder
ARBD: Alcohol-Related Birth Defects
ARND: Alcohol-Related Neurodevelopmental Disorder
CANTAB: Cambridge Neuropsychological Test Automated Battery
CD: Conduct Disorder
CMS: Children’s Memory Scale
D-KEFS: Delis-Kaplan Executive Function System
FAS: Fetal Alcohol Syndrome
FASD: Fetal Alcohol Spectrum Disorder
MVWM: Morris Virtual Water Maze
ND-PAE: Neurobehavioral Disorder Associated with Prenatal Alcohol Exposure
NES3: Neurobehavioral Evaluation System 3
NST: Neurobehavioral Screening Tool
ODD: Oppositional Defiant Disorder
pFAS: Partial Fetal Alcohol Syndrome
WISC-IV: Wechsler Intelligence Scale for Children, Fourth Edition
WMS-IV: Wechsler Memory Scale, Fourth Edition
WRAT4: Wide Range Achievement Test, Fourth Edition

## Footnotes

1 Sensitivity and specificity are measures of a binary classification test’s accuracy. Sensitivity is the probability of a positive test result among those with the condition (i.e., the percentage of individuals who are correctly identified as having the condition), while specificity is the probability of a negative test result among those without the condition (i.e., the percentage of individuals who are correctly identified as not having the condition).
